# Supplementary material for: Adverse childhood experiences and resilience among adult women: A population-based study
Source: eLife. 2022 Feb 1;11:e71770. doi: 10.7554/eLife.71770 (PMC8806181; doi:10.7554/eLife.71770)
Supplement: Transparent reporting form [file elife-71770-transrepform1.docx]

***eLife’s* transparent reporting form**

We encourage authors to provide detailed information *within their submission* to facilitate the interpretation and replication of experiments. Authors can upload supporting documentation to indicate the use of appropriate reporting guidelines for health-related research (see [EQUATOR Network](http://www.equator-network.org/%20)), life science research (see the [BioSharing Information Resource](https://biosharing.org/)), or the [ARRIVE guidelines](http://www.plosbiology.org/article/info:doi/10.1371/journal.pbio.1000412) for reporting work involving animal research. Where applicable, authors should refer to any relevant reporting standards documents in this form.

If you have any questions, please consult our Journal Policies and/or contact us: [editorial@elifesciences.org](mailto:editorial@elifesciences.org).

**Sample-size estimation**

- You should state whether an appropriate sample size was computed when the study was being designed
- You should state the statistical method of sample size computation and any required assumptions
- If no explicit power analysis was used, you should describe how you decided what sample (replicate) size (number) to use

Please outline where this information can be found within the submission (e.g., sections or figure legends), or explain why this information doesn’t apply to your submission:

Since we have continuous measures of both exposures and outcomes and a sample of >20.000 individuals, we judged this as providing sufficient power for the planned analyses and therefore did not specifically perform a formal power analysis.

**Replicates**

- You should report how often each experiment was performed
- You should include a definition of biological versus technical replication
- The data obtained should be provided and sufficient information should be provided to indicate the number of independent biological and/or technical replicates
- If you encountered any outliers, you should describe how these were handled
- Criteria for exclusion/inclusion of data should be clearly stated
- High-throughput sequence data should be uploaded before submission, with a private link for reviewers provided (these are available from both GEO and ArrayExpress)

Please outline where this information can be found within the submission (e.g., sections or figure legends), or explain why this information doesn’t apply to your submission:

We only performed the analyses on a single population with data on all relevant measures. Our results are in line with and, hence, supported by previous reports.

**Statistical reporting**

- Statistical analysis methods should be described and justified
- Raw data should be presented in figures whenever informative to do so (typically when N per group is less than 10)
- For each experiment, you should identify the statistical tests used, exact values of N, definitions of center, methods of multiple test correction, and dispersion and precision measures (e.g., mean, median, SD, SEM, confidence intervals; and, for the major substantive results, a measure of effect size (e.g., Pearson's r, Cohen's d)
- Report exact p-values wherever possible alongside the summary statistics and 95% confidence intervals. These should be reported for all key questions and not only when the p-value is less than 0.05.

Please outline where this information can be found within the submission (e.g., sections or figure legends), or explain why this information doesn’t apply to your submission:

The statistical analyses are described in detail in the Methods section (pages 10-11).

All analyses are based on participants with less than 25% missingness (N=26,198 women) and the analyses were re-done using complete cases, i.e. those with complete data on all measures of interest (N= 19,637 women), yielding comparable results. In supplementary materials, the analyses are stratified by e.g. social support and then the N is specifically noted (N=25,314 women).

P values for β and Prevalence Ratios (PR) are not reported, since confidence intervals are already informative on statistical significance. Exact p-values are reported for descriptive analyses.

(For large datasets, or papers with a very large number of statistical tests, you may upload a single table file with tests, Ns, etc., with reference to sections in the manuscript.)

**Group allocation**

- Indicate how samples were allocated into experimental groups (in the case of clinical studies, please specify allocation to treatment method); if randomization was used, please also state if restricted randomization was applied
- Indicate if masking was used during group allocation, data collection and/or data analysis

Please outline where this information can be found within the submission (e.g., sections or figure legends), or explain why this information doesn’t apply to your submission:

The ascertainment of exposure (adverse childhood experiences, ACEs) is described in the Methods section (page 6).

Three types of exposure variables were used: i) a continuous ACE-IQ total score ranging from 0 to 13 (used to assess continuous associations, and therefore with no reference group), ii) the total score was categorized (0, 1, 2, 3-4, and ≥5 ACEs), where 0 ACEs serves as the reference group and 1-≥5 ACEs represent varying degrees of ACE exposure, and iii) binary variable for each individual ACE type, coded as 0 (unexposed) and 1 (exposed).

**Additional data files (“source data”)**

- We encourage you to upload relevant additional data files, such as numerical data that are represented as a graph in a figure, or as a summary table
- Where provided, these should be in the most useful format, and they can be uploaded as “Source data” files linked to a main figure or table
- Include model definition files including the full list of parameters used
- Include code used for data analysis (e.g., R, MatLab)
- Avoid stating that data files are “available upon request”

Please indicate the figures or tables for which source data files have been provided:

The graphs (Figure 1 and Figure 2) have numerical data embedded (β or Prevalence Ratios together with confidence intervals).

Model definitions, including covariates, have been thoroughly described in the Methods section (pages 10-11).

We have uploaded an R script used for primary analyses.
